# A Two-Sample Mendelian Randomisation Analysis of the Oral Microbiome and Oral/Oropharyngeal/Tongue Cancers

**DOI:** 10.3290/j.ohpd.c_2778

**Published:** 2026-08-12

**Authors:** Leng Han, Xinxuan Wu, Beini Gong, Xun Li, Xiao Li, Zhifa Wang

**Affiliations:** a Leng Han Clinical Pathologist, Department of Pathology, The First Affiliated Hospital of Guangzhou University of Chinese Medicine, Guangzhou, Guangdong 510405, China. Conceived the study, designed and optimised the study, acquired and analysed the data, and drafted the manuscript.; b Xinxuan Wu Dentist, School of Stomatology, Jinan University, Guangzhou, Guangdong 510632, China; Department of Stomatology, General Hospital of Southern Theatre Command of PLA, Guangzhou, Guangdong 510010, China. Acquisition, analysis, and interpretation of data.; c Beini Gong Dentist, Department of Stomatology, General Hospital of Southern Theatre Command of PLA, Guangzhou, Guangdong 510010, China; Department of Stomatology of Southern Medical University, Guangzhou, Guangdong 510510, China. Analysis and interpretation of data.; d Xun Li Dentist, Department of Stomatology, General Hospital of Southern Theatre Command of PLA, Guangzhou, Guangdong 510010, China; Department of Stomatology of Southern Medical University, Guangzhou, Guangdong 510510, China. Analysed and interpreted data; advised on the study and revised the manuscript.; e Xiao Li Dentist, Department of Stomatology, General Hospital of Southern Theatre Command of PLA, Guangzhou, Guangdong 510010, China. Analysed and interpreted data; advised on the study and revised the manuscript.; f Zhifa Wang Oral and maxillofacial surgeon, Department of Stomatology, General Hospital of Southern Theatre Command of PLA, Guangzhou, Guangdong 510010, China; School of Stomatology, Southern Medical University, Guangzhou, Guangdong 510510, China. Conceived the study, designed and optimised the study, acquired and analysed the data, and drafted the manuscript. Provided guidance, obstacle removal, and interpretation of results and final approval of the version to be submitted.

**Keywords:** causal relationship, Mendelian randomisation, oral cancer, oral and oropharyngeal cancer, tongue cancer

## Abstract

**Objective:**

To assess the potential causal relationships of the oral microbiome with the risks of oral cancer, oropharyngeal cancer, and tongue cancer using two-sample Mendelian randomisation (MR) analysis, while distinguishing these from reverse causal effects of the cancers on microbial abundance.

**Methods and Materials:**

Using single-nucleotide polymorphisms as instrumental variables, we applied the MR inverse-variance-weighted approach to evaluate the effects of the dorsal-tongue and salivary microbiomes on oral, oropharyngeal, and tongue cancers. Analyses were conducted with the R package TwoSampleMR, leveraging genome-wide association study (GWAS) summary statistics from CNGBdb, the FinnGen consortium, and other sources. Sensitivity, heterogeneity, and pleiotropy assessments were performed. Additionally, reverse MR sensitivity analyses were conducted to explore the possible causal influence of cancers on the oral microbiota.

**Results:**

Using a single nucleotide polymorphism (SNP) significance threshold of *p* < 5 × 10^–6^, our large-scale MR study revealed genetically supported causal relationships between microbial taxa derived from saliva and the tongue and the risk of oral, oropharyngeal, and tongue cancers. Integrating these results, we found that both ‘s Veillonella_rogosae_mgs_2008’ and ‘s unclassified_mgs_1048’ conferred a reduced risk of oropharyngeal and tongue cancers. Sensitivity analyses based on heterogeneity tests and pleiotropy evaluations further corroborated the robustness of our findings, lending additional credibility to the conclusions.

**Conclusion:**

This study leveraged large-scale publicly available genetic data and identified significant causal relationships between the oral microbiota and cancers of the oral cavity, oropharynx, and tongue. Reverse MR analyses indicated that oral and tongue cancers may in turn alter the abundance of specific oral microbes, suggesting a potential bidirectional causal loop. Future work should integrate metagenomic data to further validate these microbiota–cancer associations.

Oral, oropharyngeal, and tongue cancers are among the most common solid tumours worldwide, with approximately half of all cases occurring in Asia.^38^ Between 1990 and 2021, the age-standardised incidence of lip/oral cavity and tongue cancer in China rose steadily; the average annual per cent change (AAPC) was 2.39 (95 % CI: 2.13–2.65) in men and 0.30 (95 % CI: 0.08 – 0.53) in women, indicating a faster increase among males.^44^ Tongue cancer accounts for roughly 40% of oral malignancies and is showing a clear trend toward earlier onset.^6^ Patients diagnosed at advanced stages have a 5-year survival rate of < 50 %, while recurrence, swallowing and speech impairment, facial disfigurement, and social stigma impose a heavy burden on daily life and mental health.^11^ Clinical efforts remain focused on ‘early screening–early diagnosis–early treatment’. For locally advanced or metastatic disease, neoadjuvant immunotherapy/targeted therapy combined with conventional surgery and chemoradiation achieves pathological response rates of 53–73% and improves survival, yet overall efficacy has reached a plateau.^12,35
^


As one of the five major human micro-ecosystems alongside those of the nose, vagina, gut and skin, the oral microbiota not only drives dental caries and periodontitis when dysbiotic, but also influences systemic disease through ‘oral–gut’, ‘oral–circulation’ and ‘oral–gut–brain’ axes.^1,5,20,25,33,37
^ Prospective cohorts have linked specific oral taxa (e.g. *Fusobacterium nucleatum* and *Porphyromonas gingivalis*) to increased colorectal cancer risk, while multi-omics studies show the oral microbiota can intensify inflammatory bowel disease activity and contribute to atherosclerosis via endotoxin-mediated inflammation.^16,43
^ Intratumoral bacterial signals in glioblastoma display species-level sequence similarity to organisms resident in the oral cavity and gut.^29^ In head-and-neck oncology, case–control and longitudinal studies consistently report elevated diversity and higher abundances of putative pathogens in oral-cancer patients, changes that coincide with p53 mutations and CpG-island hypermethylation, suggesting microbial involvement in oral carcinogenesis and progression.^24,32,41
^ Yet most evidence is cross-sectional, precluding distinction between ‘microbes drive cancer’ and ‘cancer reshapes microbes’. Small Mendelian randomisation studies have suggested a causal effect of *Porphyromonas gingivalis* on oral-cancer risk, but findings await replication.^23^ Robust, large-scale investigations are therefore required to clarify causal pathways linking the oral microbiota to cancers of the oral cavity, oropharynx and tongue.^3^


Mendelian randomisation (MR) exploits the random allocation of parental genetic variants to offspring as instrumental variables (IVs) to estimate the causal effect of an exposure on an outcome free from confounding and reverse causation.^34^ In recent years, MR has been increasingly applied to human microbiome research: for example, an MR study based on the MiBioGen consortium demonstrated that elevated abundance of intestinal ‘Bifidobacterium’ prolongs life expectancy, while another reported that *Fusobacterium nucleatum* promotes oesophageal squamous cell carcinoma by upregulating IL-6 signalling.^22,27
^ In the oral field, two-sample MR has suggested a positive causal association between oral spirochetes and pancreatic-cancer risk, yet systematic MR investigations specifically targeting oral, oropharyngeal and tongue cancers remain to be conducted.^10,48
^


Using publicly available genome-wide association study (GWAS) summary data, we conducted a two-sample MR analysis to evaluate the potential causal effects of the oral microbiota on oral cavity, oropharyngeal, and tongue cancers. Inverse-variance weighted (IVW) regression served as the primary analysis, complemented by sensitivity analyses employing MR-Egger, weighted median, weighted mode, MR-PRESSO, and HEIDI-outlier tests. Heterogeneity was assessed with Cochran’s Q statistic.^45^ This study aims to assess the potential causal relationships of the oral microbiome with the risks of oral cancer, oropharyngeal cancer, and tongue cancer, while distinguishing these from reverse causal effects of the cancers on microbial abundance, as well as to lay a population-level foundation for precision ‘microbiota-to-cancer’ intervention strategies.

## Methods and Materials

### Data Sources

Oral microbiome data were extracted from a recently published large-scale GWAS (PMID 34873157) comprising 1915 dorsal-tongue and 2984 saliva samples from 2017 healthy Chinese individuals.^8^ Summary-level GWAS data for oral cavity cancer were obtained from the FinnGen consortium; all participants were of European ancestry and provided informed consent, including 1,614 oral cavity cancer cases and 378,749 controls. GWAS summary statistics for oral and oropharyngeal cancer were downloaded from the IEU OpenGWAS project, encompassing 839 cases and 372,016 controls of European ancestry. Tongue-cancer GWAS summary data were retrieved from the GWAS Catalog, covering 144 cases and 456,132 controls of European ancestry.

### MR Analyses

Using the TwoSampleMR R package (version 0.6.3), we conducted a two-sample MR analysis to assess the causal relationships between the oral microbiota and cancers of the oral cavity, oropharynx, and tongue.^24^ MR employs genetic variants as proxies for the exposure; hence, valid instrumental variables (IVs) must satisfy three key assumptions: (1) the genetic variants are robustly associated with the exposure; (2) the variants are independent of potential confounders between the exposure and the outcome; and (3) the variants influence the outcome only through the exposure (i.e. no horizontal pleiotropy).

#### Selection of instrumental variables (IVs)

For the forward MR analysis, we selected single nucleotide polymorphisms (SNPs) associated with oral microbiota at genome-wide significance thresholds of *p* < 5 × 10^–8^, *p* < 5 × 10^–7^ and *p* < 5 × 10^–6^. For the reverse MR analysis, SNPs associated with the outcome trait at p < 5 × 10^–6^ were used as IVs. Linkage-disequilibrium (LD) clumping was then performed with PLINK v1.90, using a 10 000-kb window and an r^2^ threshold of 0.001; LD r^2^ was estimated with the 1000 Genomes Project as the reference panel.^4,31
^ For each IV, we calculated the proportion of phenotypic variance explained (PVE) and the F-statistic (F = β^2^/SE^2^) to assess instrument strength and guard against weak-instrument bias. SNPs with F < 10 and palindromic SNPs were excluded, leaving the final set of IVs for subsequent analyses.

#### Selection of causal estimation methods for oral-microbiota traits on cancers

Perform the following MR analyses: use oral-cancer microbiota data as exposure and oral cancer, oropharyngeal cancer, and tongue cancer as outcomes in forward Mendelian randomisation (forward MR). Apply five methods: inverse-variance weighted (IVW), MR-Egger, weighted median, simple mode, and weighted mode.^13,18,30
^ Obtain effect sizes and *p* values for each method (*p* < 0.05 indicates a positive result), with IVW as the primary estimate and the remaining methods as supporting evidence.

#### Heterogeneity test

Heterogeneity was tested with both MR-Egger and IVW regression; the respective Q values were used to judge whether heterogeneity was present, and *p* > 0.05 was taken to indicate its absence.^4,30
^ The validity of the selected instrumental variables (IVs) was appraised by examining how much the causal estimates differed across individual IVs. Specifically, the function mr_heterogeneity in the R package TwoSampleMR was used to compute Cochran’s Q statistic:

Q = Σj wj (βĵ – β̂ )²,

where βĵ is the causal estimate obtained with the j-th IV, wj is its corresponding weight, and β̂ is the pooled estimate derived from IVW or MR-Egger regression.

#### Pleiotropy test

We used MR-Egger regression to test the pleiotropy level of the MR findings.^4^ A statistically significant deviation of the Egger intercept from zero indicates directional (horizontal) pleiotropy, i.e. the instrumental variants influence the outcome independently of the exposure, violating the core assumption of Mendelian randomisation. In addition, MR-PRESSO was applied to screen for pleiotropy; *p* values were derived from 1000 permutations, and estimates with *p* > 0.05 were considered free of horizontal pleiotropy.^39^


##### Reverse analyses of the causal computational method selection for cancer on oral-microbiota traits

To investigate whether oral cancer, oropharyngeal cancer, and tongue cancer have a causal effect on the oral microbiota, we used the IVW, MR-Egger, weighted median, simple mode, and weighted mode methods implemented in the TwoSampleMR R package to test the significance of the causal associations between these phenotypes.

##### Reverse MR sensitivity analysis

Heterogeneity was assessed using both MR-Egger and IVW methods, and the presence of heterogeneity was determined from the Q values produced by each approach. To guard against directional pleiotropy, we employed the commonly used MR-Egger regression; a significant intercept term was taken as evidence of horizontal pleiotropy. We further applied the MR pleiotropy residual sum and outlier (MR-PRESSO) method to identify and remove outlying variants that contribute to pleiotropic bias.

### Statistical Analyses

All statistical analyses were performed using R software, with ‘TwoSample MR’ and ‘MR-PRESSO’ packages specifically designed for MR analyses. The significance threshold for the MR analyses was set at *p* < 0.05. Results were visualised using forest and bubble plots to illustrate the effect sizes and confidence intervals (CI) of the associations between microbiota taxa and OOPSCC risk. These visualisations facilitate the interpretation of the findings and highlight the most significant associations.

## Results

### Oral Microbiota Instrumental Variable

As described in the Methods and Materials, a significance threshold of 5 × 10^–8^ was used to select SNPs associated with oral-microbiota phenotypic traits as instrumental-variable (IV) loci for the exposure, yielding IVs for 116 oral taxa (dorsal-tongue: 65; saliva: 51) that were carried forward to the MR analysis. At the 5 × 10^–7^ threshold, IVs for 673 taxa (dorsal-tongue: 377; saliva: 296) were extracted, and at the 5 × 10^–6^ threshold IVs for 2296 taxa (dorsal-tongue: 1203; saliva: 1093) were obtained.

### Causal Effect Estimates of Oral-Microbiota Phenotypes on Oral Cancer

At SNP significance thresholds of *p* < 5 × 10^–8^ and *p* < 5 × 10^–7^, no causal association was detected between oral-microbiota traits and oral cancer. Relaxing the threshold to *p* < 5 × 10^–6^ identified 30 microbiota phenotypes significantly associated with oral cancer (*p* < 0.05; Fig 1). Eleven of these phenotypes originated from saliva samples and 19 from dorsal-tongue samples. Specifically, taxa such as s unclassified_mgs_2249 (OR = 2.060, *p* = 0.009), s unclassified_mgs_384 (OR = 1.843, *p* = 0.02), s unclassified_mgs_1990 (OR = 2.032, *p* = 0.004), and g unclassified_mgs_1111 (OR = 1.918, *p* = 0.02) were associated with an increased risk of oral cancer, whereas s unclassified_mgs_3390 (OR = 0.663, *p* = 0.04), s unclassified_mgs_1980 (OR = 0.663, *p* = 0.04), s Simonsiella_muelleri_mgs_3058 (OR= 0.669, *p* = 0.03), and s unclassified_mgs_1538 (OR = 0.602, *p* = 0.04) showed a protective effect. Further details are provided in Supplementary Tables 1 and 2.

**Fig 1 Fig1:**
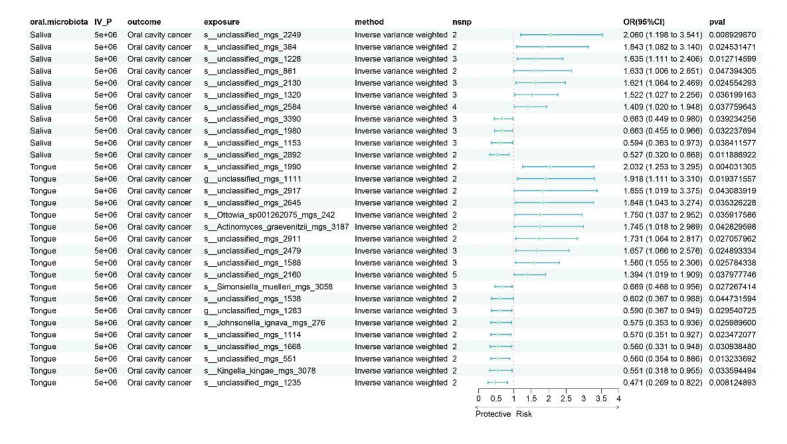
Significant causal effects of oral-microbiota phenotypes on oral cancer. This figure presents the effect estimates (odds ratios) and 95% confidence intervals for the associations between oral microbiota taxa and oral cancer risk using MR-IVW methods.

Sensitivity analyses of these findings are summarised in Supplementary Table 3. Heterogeneity tests indicated no significant heterogeneity among the SNPs contributing to the 30 microbiota traits (*p* > 0.05). Pleiotropy tests were also non-significant (*p* > 0.05), supporting the assumption that the selected SNPs influence oral cancer through the oral microbiota rather than via alternative pathways, thereby confirming the reliability and lack of bias in our results.

### Causal Effect Results of Oral-Microbiota Phenotypes on Oropharyngeal Cancer

Setting the SNP threshold at *p* < 5 × 10^–8^ yielded no causal associations between oral microbiota and oral-oropharyngeal cancer. When the threshold was relaxed to *p* < 5 × 10^–7^, two taxa – one from saliva and one from dorsal-tongue – were identified (Fig 2). Two oral-microbiota phenotypes showed significant effects on cancer risk (*p* < 0.05): s unclassified_mgs_2259 (OR= 1.002, *p* = 0.02) increased risk, and s unclassified_mgs_386 (OR = 0.999, *p* = 0.03) decreased risk. Lowering the threshold to *p* < 5 × 10^–6^ identified 34 significant phenotypes (18 saliva-derived, 16 dorsal-tongue-derived). Taxa that increased risk included: s unclassified_mgs_2743 (OR = 1.002, *p* = 0.02), s unclassified_mgs_1602 (OR = 1.002, *p* = 0.048), s *Fusobacterium_periodonticum*_C_mgs_3022 (OR = 1.002, *p* = 0.002), s *Streptococcus_oralis*_Z_mgs_3165 (OR = 1.003, *p* = 0.04). Protective taxa included: s unclassified_mgs_516 (OR = 0.998, *p* = 0.03), s unclassified_mgs_2276 (OR = 0.998, *p* = 0.04), s unclassified_mgs_2858 (OR = 0.998, *p* = 0.02), s unclassified_mgs_1705 (OR = 0.998, *p* = 0.04). Notably, s unclassified_mgs_386 remained protective at both *p* < 5 × 10^–7^ and *p* < 5 × 10^–6^, and s unclassified_mgs_2858 was protective in both saliva and dorsal-tongue samples. Full causal effect estimates are provided in Supplementary Tables 4 and 5.

**Fig 2 Fig2:**
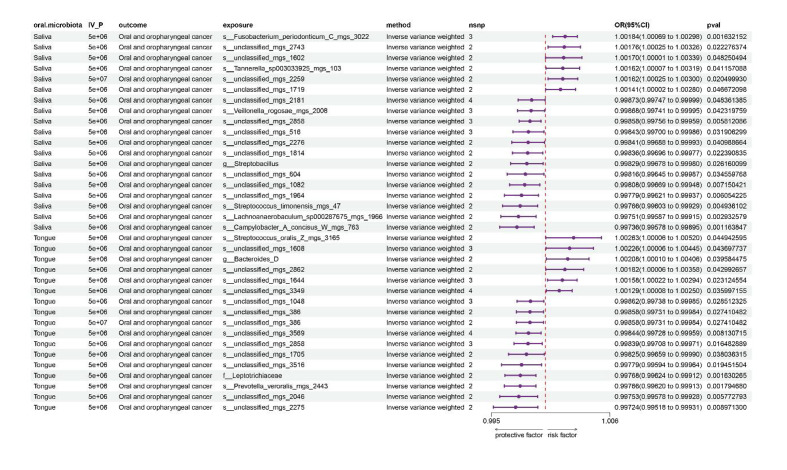
Significant causal effects of oral microbiota phenotypes on oral and oropharyngeal cancer. This figure presents the effect estimates (odds ratios) and 95% confidence intervals for the associations between oral microbiota taxa and oral and oropharyngeal cancer risk using MR-IVW methods.

Sensitivity analyses (Supplementary Table 6) showed no evidence of heterogeneity among the 34 sets of SNPs (*p* > 0.05). Pleiotropy tests were likewise non-significant (*p* > 0.05), indicating that the selected SNPs influence oral-oropharyngeal cancer through the identified oral-microbiota traits and that our estimates are unbiased.

### Causal Effect Estimates of Oral-Microbiota Phenotypes on Tongue Cancer

Results showed no causal association between oral microbiota and oral cancer selected at SNP threshold *p* < 5 × 10^–8^. When the SNP threshold was relaxed to *p* < 5 × 10^–7^, two taxa from the saliva category were identified (Fig 3). Specifically, g unclassified_mgs_377 (OR = 4.38, *p* = 0.04) increased the risk of tongue cancer, whereas s unclassified_mgs_1590 (OR= 0.12, *p* = 0.006) decreased it. At *p* < 5 × 10^–6^, 28 oral-microbiota phenotypes showed significant associations with tongue cancer (p < 0.05); 13 originated from saliva and 15 from dorsal-tongue samples. Taxa that increased risk included s unclassified_mgs_1310 (OR = 4.39, p = 0.02), g unclassified_mgs_377 (OR = 4.38, *p* = 0.04), and s *Eikenella corrodens*_mgs_821 (OR = 3.07, *p* = 0.046). Protective taxa comprised s unclassified_mgs_2776 (OR = 0.27, *p* = 0.03), s *Neisseria* sp000186165_mgs_2832 (OR = 0.22, *p* = 0.04), s unclassified_mgs_1048 (OR = 0.26, *p* = 0.048), and s unclassified_mgs_2210 (OR = 0.25, *p* = 0.049).

**Fig 3 Fig3:**
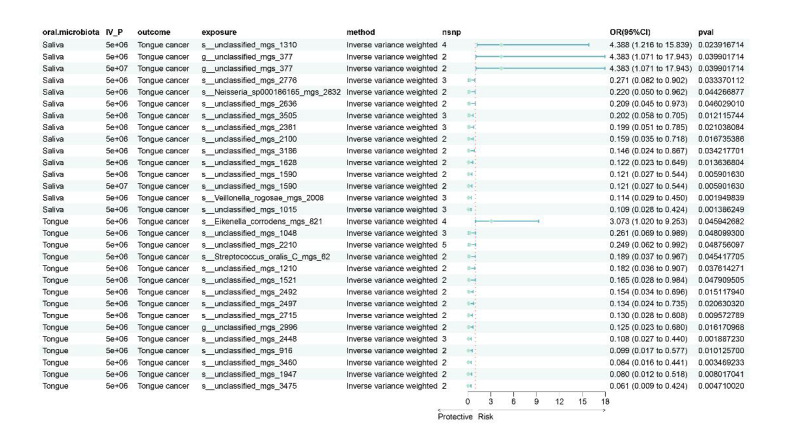
Significant causal effects of oral microbiota phenotypes on tongue cancer. This figure presents the effect estimates (odds ratios) and 95% confidence intervals for the associations between oral microbiota taxa and tongue cancer risk using MR-IVW methods.

Notably, g unclassified_mgs_377 consistently increased tongue-cancer risk at both *p* < 5 × 10^–7^ and *p* < 5 × 10^–6^, while s unclassified_mgs_1590 consistently decreased it. Further details are provided in Supplementary Tables 7 and 8. Sensitivity analyses are presented in Supplementary Table 9. Homogeneity tests revealed no heterogeneity among the SNPs for any of the 28 microbiota traits (*p* > 0.05). Pleiotropy tests were also non-significant (*p* > 0.05), indicating that these SNPs influence tongue cancer through the oral microbiota and that our findings are robust and unbiased.

Finally, s *Veillonella rogosae*_mgs_2008 and s unclassified_mgs_1048 were associated with reduced risk of both oropharyngeal and tongue cancers (Fig 4).

**Fig 4 Fig4:**
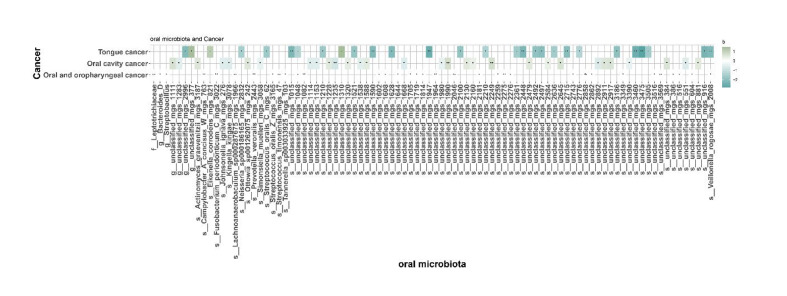
Summary of causal associations between the oral microbiota and cancers of the oral cavity, oropharynx, and tongue.*: *p* < 0.05, **: *p* < 0.01, ****p* < 0.005.

### Causal Effect Results of Oral Cancer, Oropharyngeal Cancer, and Tongue Cancer on the Oral Microbiota

As described in the Methods and Materials, the IV significance threshold for oral and oropharyngeal cancer was set to 5 × 10^–6^, with filters of minor allele frequency (MAF) > 1% and F-statistic > 10. After screening, ^11^ independent SNPs remained; details are provided in Supplementary Table 10.

Thirty-four oral-microbiota-related phenotypes showing significant causal associations were selected as outcome variables for two-sample Mendelian randomisation, and *p* values for the corresponding models were calculated; the results are presented in Supplementary Tables 11 and 12. The IVW method was adopted as the primary criterion for statistical significance, with the threshold set at *p* < 0.05. No significant causal effects of oral/oropharyngeal cancer on any of the 34 microbiota-related phenotypes were observed.

For oral cavity cancer and tongue cancer, the IV significance threshold was likewise set to 5 × 10^–6^, applying the same filters (MAF > 1 %, F > 10). After quality control, 19 and 10 independent SNPs remained, respectively; detailed information is given in Supplementary Tables 13 and 14. Oral-microbiota phenotypes with significant causal associations were then used as outcomes in two-sample MR analyses; the corresponding *p* values are reported in Supplementary Tables 15 to 18.

Again, IVW was the primary method, with significance defined as *p* < 0.05. As shown in Figure 5, oral cavity cancer exhibited significant reverse causal effects on s unclassified_mgs_2249 (OR = 1.07, *p* = 0.045) and s unclassified_mgs_2584 (OR = 1.09, *p* = 0.01), whereas tongue cancer showed significant reverse causal effects on s unclassified_mgs_3475 (OR= 0.98, *p* = 0.007) and s unclassified_mgs_1048 (OR = 0.98, *p* = 0.02).

**Fig 5 Fig5:**
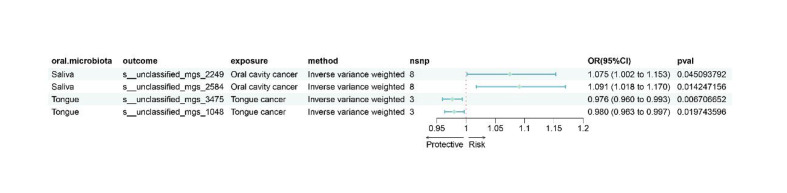
Results of the reverse MR analysis. Oral cavity cancer exhibited significant reverse causal effects on s unclassified_mgs_2249 (OR = 1.07, *p* = 0.045) and s unclassified_mgs_2584 (OR = 1.09, *p* = 0.01), whereas tongue cancer showed significant reverse causal effects on s unclassified_mgs_3475 (OR= 0.98, *p* = 0.007) and s unclassified_mgs_1048 (OR= 0.98, *p* = 0.02).

Sensitivity analyses for these findings are summarised in Supplementary Table 19. Heterogeneity tests indicated no significant heterogeneity among the SNPs for the four identified microbiota traits (*p* > 0.05). Pleiotropy tests likewise yielded non-significant results (*p* > 0.05), supporting the notion that these SNPs influence oral cavity and tongue-cancer risk through the oral microbiota. Collectively, these analyses indicate that our MR estimates are reliable and free from substantial bias.

## Discussion

In this study, we leveraged publicly available summary statistics from the latest large-scale GWAS of the oral microbiota, together with oral-cancer data from the FinnGen consortium, oropharyngeal-cancer data from the IEU OpenGWAS project, and tongue-cancer data from the GWAS Catalog, to perform two-sample MR analyses and assess causal relationships between the oral microbiota and these three malignancies. Forward MR indicated that 13 oral taxa – among them s unclassified_mgs_3390 (*Solobacterium*), s unclassified_mgs_1980 (*Aggregatibacter*) and s *Simonsiella_muelleri*_mgs_3058 (*Simonsiella*) – significantly decrease the risk of oral cancer, whereas 17 taxa – including s unclassified_mgs_2249 (*Campylobacter*) and g unclassified_mgs_1111 (*Saccharimonadaceae*) – increase that risk. For oropharyngeal cancer, 12 taxa such as s *Fusobacterium_periodonticum*_C_mgs_3022 (*Fusobacterium*), s unclassified_mgs_2259 (*Granulicatella*) and s unclassified_mgs_3349 (*Haemophilus*) conferred higher risk, while 22 taxa – e.g. s unclassified_mgs_2276* (*Prevotella*), s unclassified_mgs_1082 (*Solobacterium*) and s unclassified_mgs_1964 (*Streptococcus*) – were protective. Regarding tongue cancer, three taxa (g unclassified_mgs_377 [*Saccharimonadaceae*], s *Eikenella_corrodens*_mgs_821 [*Eikenella*] and s unclassified_mgs_1310 [UBA3738]) increased risk, whereas 25 taxa – such as s *Neisseria*_sp000186165_mgs_2832 (*Neisseria*), s unclassified_mgs_2210 (*Streptococcus*) and s unclassified_mgs_2361 (*Capnocytophaga*) – reduced risk. Reverse Mendelian randomisation (MR) analyses further suggested potential associations between oral cancer and *Campylobacter abundance*, as well as between tongue cancer and both *Solobacterium* and *Saccharimonadaceae*. However, due to limited statistical power, no significant genetically predicted effects of cancers on the microbiome were detected; therefore, a bidirectional causal relationship cannot be directly inferred. For taxa with low statistical power, negative results may reflect insufficient power rather than a true absence of association.

A growing body of research has established a causal link between the oral microbiota and oral cancer.^24,40,41
^ For example, ‘Prevotella’ has been shown to increase oral-cancer risk, a finding that aligns with our own results; however, the precise mechanism remains unclear.^41,42
^ One hypothesis is that *Prevotella* activates the IL-6/STAT3 inflammatory axis, thereby promoting carcinogenesis. *Streptococcus* and *Veillonella* have likewise been reported to exhibit a positive causal relationship with oral cancer^15,24
^ yet in our data the strain s unclassified_mgs_1538 (*Streptococcus*) and s *Kingella_kingae*_mgs_3078 (family *Neisseriaceae*) were associated with a reduced risk. Given differences in genetic background, dietary habits, and living environments among study populations, together with strain-level functional variations – whereby different species within the same genus may exert opposing functions (e.g. pathogenic versus commensal subtypes of *Fusobacterium nucleatum*) – these disparities may ultimately manifest as functional heterogeneity among strains or subtypes within the same genus, encompassing differences in gene content, metabolic outputs, and host-interaction modes, and thereby potentially leading to diametrically opposing oncogenic or onco-protective effects.^9,21
^ Illustratively, s unclassified_mgs_1668 (f *Saccharimonadaceae*, g TM7x) and s unclassified_mgs_1235 (f *Saccharimonadaceae*, g TM7x) decreased oral-cancer incidence, whereas g unclassified_mgs_1111 (f *Saccharimonadaceae*) and s unclassified_mgs_2917 (f *Saccharimonadaceae*, g TM7x) increased it. A similar pattern was observed for oropharyngeal cancer: s unclassified_mgs_2858 (f *Fusobacteriaceae*, g *Fusobacterium*) conferred protection, while s *Fusobacterium_periodonticum*_C_mgs_3022 (f *Fusobacteriaceae*, g *Fusobacterium*) elevated risk. Disentangling these opposing ‘pro- vs anti-cancer’ effects of individual strains will therefore require future studies that integrate population-level epidemiology with deep genomics and machine-learning pipelines – closing the loop ‘from cohorts to mechanisms, from metagenomes to single isolates, and from association to causal intervention’.^26^


The oral microbiota, the second most abundant microbial community in the human body after the gastrointestinal tract, maintains homeostasis that is essential for normal physiological, metabolic and immune functions.^19,40
^ Alterations of the oral microbiome have been observed in bacteraemia, diabetes, HIV-related immunodeficiency, systemic lupus erythematosus and gastrointestinal malignancies, and dysbiotic oral flora with tumour-promoting potential has also been documented in oral cancer patients – including pronounced microbiome shifts in areca-nut-chewing–associated oral submucous fibrosis and related oral cancers.^2,16,46
^ Multiple factors can disrupt oral microbial equilibrium, including diet, oral hygiene, smoking, alcohol intake, medications and climate change.^36^ In addition, surgery, radiotherapy and chemotherapy all modify the composition and abundance of oral flora, while remodelling of the oral microbiome in turn modulates the efficacy of chemoradiotherapy and immune-targeted therapy for oral cancer.^28^ Moreover, as the entry point of the digestive tract, the oral microbiota influences gut microbiota and exhibits causal links to intestinal disorders such as inflammatory bowel disease and colorectal cancer.^14,48
^


Oral squamous cell carcinoma (OSCC) is the most common subtype of oral cancer.^24^ After conventional treatment, patients often show a persistently elevated abundance of potentially pathogenic organisms in saliva, together with a long-lasting reduction in salivary microbial α-diversity and increased variability that can persist for years.^17^ Consequently, patients with oral or tongue cancer require intensified postoperative oral care; interventions such as probiotics or prebiotics, artificial saliva, or scientifically tailored diets may improve patient outcomes by mitigating oral dysbiosis.^28^ This concept is further supported by the finding that metastatic renal-cell carcinoma patients who received the immunotherapy plus oral CBM588 – a live bifidobacterial product – exhibited significantly longer progression-free survival and higher response rates.^7^ These observations underscore the broader link between the human microbiome and longevity: gut microbes with protective properties can increase the probability of living longer, and the oral microbiome is similarly implicated; centenarians show no major genetic divergence in their oral microbiome but do display reduced gut microbial diversity.^27,47
^


However, this study also has several limitations. First, the oral microbiota data were derived from an East Asian population, whereas the data for oral, oropharyngeal, and tongue cancers were obtained from European populations. Given the differences in lifestyle and dietary habits between Asian and European populations, the oral microbial composition is likely to differ as well; consequently, the results of this two-sample MR analysis may not fully reflect a true causal relationship.^10^ Second, most of the bacterial taxa examined in this study were identified only at the genus level. Future studies should employ metagenomic sequencing and other advanced technologies to achieve precise characterisation at the genus, species, or even subspecies level, thereby enabling a more accurate elucidation of the causal relationship between the oral microbiota and oral malignancies and providing potential targets for the clinical prevention and treatment of these cancers.

## Conclusion

In summary, leveraging large-scale publicly available genetic data, this study demonstrates a significant causal relationship between the oral microbiota and cancers of the oral cavity, oropharynx, and tongue. Certain microbial taxa increase risk, whereas others confer protection. Reverse MR analyses further indicate that oral and tongue cancers may influence the abundance of specific oral microbes, suggesting the possibility of bidirectional causality. Future work integrating metagenomic approaches is warranted to validate these microbiome-cancer associations.

### Acknowledgements

### Supplementary Tables

To download the supplementary tables, please visit the Open Science Framework (OSF) website (https://osf.io/uvj8p/files/pvwxd).

#### Funding declaration

The present study was funded by the Natural Science Foundation of Guangdong Province of China (2024A1515220159), the Science and Technology Planning Project of Guangzhou city, China (2025A03J3186), the Joint Logistics Support Force ‘Three-Talent One-Team’ Support Programme of PLA (514319) and the Open Research Project of the National Key Laboratory of Oral and Maxillofacial Reconstruction and Regeneration of Air Force Military Medical University (2024KB08).

#### Clinical trial number

Not applicable.

#### Consent to publish declaration

All authors have read and approved the content, and agreed to submit for consideration for publication in the journal.

#### Consent to participate declaration

Not applicable.

#### Ethics declaration

Not applicable.

#### Data availability declaration

The data set(s) supporting the conclusions of this article are included within the article and its additional files.

#### Competing interest declaration

The authors declare that they have no known competing financial interests or personal relationships that could have appeared to influence the work reported in this article.

## References
